# Salidroside Attenuates Denervation-Induced Skeletal Muscle Atrophy Through Negative Regulation of Pro-inflammatory Cytokine

**DOI:** 10.3389/fphys.2019.00665

**Published:** 2019-06-25

**Authors:** Changyue Wu, Longhai Tang, Xuejun Ni, Tongtong Xu, Qingqing Fang, Lai Xu, Wenjing Ma, Xiaoming Yang, Hualin Sun

**Affiliations:** ^1^Key Laboratory of Neuroregeneration of Jiangsu and Ministry of Education, Jiangsu Clinical Medicine Center of Tissue Engineering and Nerve Injury Repair, Co-innovation Center of Neuroregeneration, Nantong University, Nantong, China; ^2^School of Medicine, Nantong University, Nantong, China; ^3^Departments of Blood Component Preparation, Suzhou Blood Center, Suzhou, China; ^4^Departments of Ultrasound, Affiliated Hospital of Nantong University, Nantong, China

**Keywords:** denervation, muscle atrophy, inflammation, salidroside, interleukin 6

## Abstract

Skeletal muscle atrophy is associated with pro-inflammatory cytokines. Salidroside is a biologically active ingredient of Rhodiola rosea, which exhibits anti-inflammatory property. However, there is little known about the effect of salidroside on denervation-induced muscle atrophy. Therefore, the present study aimed to determine whether salidroside could protect against denervation-induced muscle atrophy and to clarify potential molecular mechanisms. Denervation caused progressive accumulation of inflammatory factors in skeletal muscle, especially interleukin 6 (IL6) and its receptor, and recombinant murine IL6 (rmIL6) local infusion could induce target muscle atrophy, suggesting that denervation induced inflammation in target muscles and the inflammation may trigger muscle wasting. Salidroside alleviated denervation-induced muscle atrophy and inhibited the production of IL6. Furthermore, the inhibition of phosphorylation of signal transducer and activator of transcription 3 (STAT3), and the decreased levels of suppressor of cytokine signaling (SOCS3), muscle RING finger protein-1 (MuRF1), atrophy F-box (atrogin-1), microtubule-associated protein light chain 3 beta (LC3B) and PTEN-induced putative kinase (PINK1) were observed in denervated muscles that were treated with salidroside. Finally, all of these responses to salidroside were replicated in neutralizing antibody against IL6. Taken together, these results suggest that salidroside alleviates denervation-induced inflammation response, thereby inhibits muscle proteolysis and muscle atrophy. Therefore, it was assumed that salidroside might be a potential therapeutic candidate to prevent muscle wasting.

## Introduction

Skeletal muscle is a very important organ, which maintains many important functions, such as body movement, breathing, and glucose homeostasis (Powers et al., 2016; Qiu et al., 2018), and it is a plastic organ that is maintained according to physiological and pathological conditions. Muscle hypertrophy occurs during development and in response to mechanical overload and muscle atrophy results from denervation, aging, starvation, cancer, AIDS, diabetes, bed rest, or chronic heart failure (Schiaffino et al., 2013). Excessive loss of muscle mass is a major reason for morbidity and mortality in numerous diseases and it is associated with poor prognosis (Glass, 2003; Bonaldo and Sandri, 2013), which not only impairs the quality of life, but also bring the economic and social burden for families and communities (Qiu et al., 2018). However, the treatment of skeletal muscle wasting remains an unresolved challenge to this day. Therefore, elucidating its molecular basis and developing therapeutics to prevent or even reverse the atrophic process has become the focus of current research (Qiu et al., 2018).

The maintainment of the normal structure and function of skeletal muscle owes much to the stability of muscle microenvironment, composed of muscle stem cells, motoneurons, interstitial cells and a variety of secretory factors (Madaro et al., 2018). Once the muscle microenvironment is perturbed, the ubiquitin-proteasome system (UPS) and the autophagy-lysosome system (ALS) will be activated and accelerate muscle atrophy (He et al., 2013; Ratti et al., 2015; Talbert and Guttridge, 2016). Inflammatory cytokines, important components of muscle microenvironment, such as tumor necrosis factor alpha (TNFα) and interleukin-6 (IL6) are important mediators of catabolic responses such as protein proteolysis (Le et al., 2014; Costamagna et al., 2015; Londhe and Guttridge, 2015; Ma et al., 2018). Loss of muscle mass is frequently associated with elevated pro-inflammatory cytokines. For example, patients affected by cachectic cancer, diabetes, obesity, chronic renal or heart failure display increased pro-inflammatory cytokines, such as TNF-α, IL1, and IL6, which are associated with decreased protein synthesis and enhanced protein breakdown (Valerio et al., 2006; Lumeng and Saltiel, 2011; Molanouri Shamsi et al., 2014; Costamagna et al., 2015; Xu et al., 2018). Indeed, muscle wasting, accompanied by excessive protein breakdown, can be prevented by treating tumor-bearing animals with antibodies against pro-inflammatory cytokines (Strassmann et al., 1993). Muscle cells are a source of IL6, which, unlike other cytokines, has a unique property of exerting both pro and anti-inflammatory (Londhe and Guttridge, 2015). Low level of IL6, produced in a contracting skeletal muscle after prolonged exercise, is therefore considered as a myokine, which promotes muscle stem cell proliferation and mediates muscle hypertrophy (Serrano et al., 2008; Munoz-Canoves et al., 2013). In cases of muscle injury, IL6 levels dramatically increase and trigger muscle wasting and chronic inflammation (Londhe and Guttridge, 2015). Furthermore, overexpressed IL6 or administration of high doses IL6 caused severe muscle atrophy due to the activation of the proteolytic pathway. Blockade of IL6 signaling reversed the muscular changes and reduced muscle atrophy (Munoz-Canoves et al., 2013). However, the role of pro-inflammatory cytokines in denervation-induced muscle atrophy remained unclear. In current study, a variety of pro-inflammatory cytokines were observed in muscles after denervation, which might also play crucial role in muscle atrophy. Given that inflammation exacerbates muscle atrophy, therapeutic drugs or countermeasures that suppress skeletal muscle inflammation and improve muscle microenvironment might be useful for preventing denervation-induced skeletal muscle atrophy.

Salidroside is a biologically active ingredient of Rhodiola rosea, which exhibits anti-inflammatory, anti-oxidative, and anti-apoptotic properties (Lai et al., 2015; Sun et al., 2018). Salidroside attenuated the infiltration of inflammatory cells and the expression of IL6 through suppressing the phosphorylation of STAT3 signaling pathway in LPS induced inflammation (Li et al., 2013; Yan and Choi, 2014; Qi et al., 2016; Wu et al., 2016). Salidroside also reduced the expression of IL6 in serum, and then exerts strong favorable cardioprotective function on myocardial I/R injury (Zhu et al., 2015). However, it is not clear whether salidroside can prevent denervation-induced skeletal muscle atrophy through suppressing inflammation.

In the present study, we for the first time demonstrated that denervation caused progressive accumulation of inflammatory factors in skeletal muscle, and salidroside alleviated skeletal muscle atrophy through reducing denervation-induced muscle inflammation. Salidroside may be a potential therapeutic candidate to prevent muscle wasting. These results uncover a new application for salidroside.

## Materials and Methods

### Animal Treatment

This study was carried out in accordance with the recommendations of the Institutional Animal Care and Use Committee of Nantong University. The protocol was approved by the Institutional Animal Care and Use Committee of Nantong University. Male SD rats (2 months old) were purchased from Laboratory Animal Center of Nantong University, Jiangsu, China. There are ten rats in each group. Rats were housed in standard cages in a room at 23°C, 50% relative humidity, and in a 12-h light/12-h dark cycle. Animals in experimental groups were subjected to unilateral sciatic nerve transection under anesthesia as described previously (Qiu et al., 2018), followed by daily intraperitoneal injection of saline (100 μL; Den group), salidroside (20mg/kg; Sigma-Aldrich, Shanghai, China) in saline (Den+Sal group), or IL6 neutralizing antibody (1.4 mg/week) in saline (Den+anti-IL6 group), respectively. Animals in normal control group received sham-operation and then injected with the same amount of saline daily (Ctrl group). To model the sustained high levels of IL6 in muscles, we implanted osmotic pumps delivering recombinant murine IL6 in normal rats. Briefly, Osmotic minipumps (ALZET model 2002; ALZA Corp, Cupertino, CA, United States) were subcutaneously implanted to infuse IL6 (Beyotime, Haimen, China) to TA muscle via a catheter (0.006 in. inner diameter, ALZA Corp, Cupertino, CA, United States) at 4.5 pg/muscle/h. The dose of IL-6 selected for this study was based on recent observations of IL-6-induced skeletal muscle atrophy (Haddad et al., 2005). Only saline-infused rats served as control group. After 14 d, rats were anesthetized and tissues were removed, weighed, and snap-frozen in liquid nitrogen before storing at −80°C.

### Microarray Analyses

The study of the differential gene expression profiling was carried out with an Agilent SurePrint-G3 Rat GE microarray kit (8 × 60K, Design ID: 028279). RNA was extracted from the skeletal muscles, harvested at different times (0 h, 0.25 h, 0.5 h, 3 h, 6 h, 12 h, 24 h, 3 d, 7 d, 14 d, 21 d, and 28 d after sciatic nerve transection in rat), using RNeasy Mini Kit (Qiagen, Valencia, CA, United States) according to the manufacturer’s instructions (Qiu et al., 2018). Microarray analysis was performed on an Agilent Gene Chip platform and scanned by Agilent Scanner G2505C (Agilent Technologies). Data were extracted using Agilent Feature Extraction Software (version 10.7.1.1, Agilent Technologies), and normalized by Genespring Software (version 13.1, Agilent Technologies). The gene expression dataset is available on EBI and the accession number is E-MTAB-8009.

### RT-PCR

Total RNA was extracted using the RNeasy kit (Qiagen, Valencia, CA, United States), cDNA was synthesized using the first-strand cDNA synthesis kit with oligo dT primers (Invitrogen, Carlsbad, CA, United States), and RT-PCR was performed (Opticon real-time PCR; MJ Research, Waltham, MA, United States). The RT-PCR conditions were as follows: 42°C for 20 min and then 40 cycles at 95°C for 5 min, 94°C for 20 s, and 72°C for 42 s. The melting curve was run at 65 to 95°C. Relative expression was calculated from cycle threshold values corrected for ACTB [Ct; relative expression = 2^(sample Ct − ACTB*Ct*)^]. The primers were as follows: rat IL6r (NM_017020) 5′- GGCAACCTTAGTGCTCAT-3′, 5′- CTGTCTGCTCCAGCTTGTTA-3′; rat STAT3 (NM_012747) 5′- GTCTGAATTAAGGGCAGTGAG-3′, 5′- CAGGGAAGGGAGAGCAATGA-3′; rat JAK2 (NM_031514) 5′- GAGCTACTGAAGAACAACGG-3′, 5′- TGAAAGAGGGACGTTGGTTGA-3′; rat SOCS3 (NM_053565) 5′- TCTCTCCTCCAACGTGGCTA-3′, 5′- GTCCAGGAACTCCCGAAT-3′; rat ACTB (NM_031144.2) 5′- CCACCATGTACCCAGGCATT-3′, 5′- CGGACTCATCGTACTCCTGC-3′.

### Western Blot Analysis

Western blot analysis was performed as described previously (Sun et al., 2014). The TA muscles were homogenized in a radio immunoprecipitation assay buffer (50 mM Tris–HCl pH 7.4, 5 mM EDTA, 150 mM NaCl, 1% sodium deoxycholate, 1% Nonidet P-40, 50 mM NaF, 0.1% SDS/1% aprotinin and 0.1 mM Na_3_VO_4_) and quantified with Bio-Rad Protein Assay Kit (Bio-Rad, Hercules, CA, United States). Equal amounts of total protein per lane were subjected to SDS-PAGE, and transferred onto PVDF (Millipore Corp), which were blocked with 5% nonfat dry milk in Tris-buffer saline (TBS) followed by incubation with primary antibodies: rabbit anti-phospho-STAT3 (Tyr705) and anti-STAT3 polyclonal antibodies (1:2000; Invitrogen Antibodies, Waltham, MA, United States), rabbit anti-LC3B, anti-PINK1 and anti-SOCS3 polyclonal antibodies (1:2000; LifeSpan BioSciences, Seattle, WA, United States), rabbit anti-MAFbx (1:3000; Abcam, Cambridge, United Kingdom), rabbit anti-MuRF-1 (1:3000; R&D Systems, Minneapolis, MN, United States) and mouse anti-tubulin (1:3000; Santa Cruz, Santa Cruz, CA, United States) at 4°C overnight. Then, the membrane was probed with the appropriate horseradish peroxidase-coupled secondary antibody. Immunoactive bands were visualized by enhanced chemiluminescence (Thermo Scientific, Park Ellisville, MO, United States). Densitometry analysis was determined by scanning immunoreactive bands, and intensity values were obtained for further normalization against loading control.

### Muscle Fiber CSA Size

The fiber CSA of TA muscles was detected by using laminin staining. Briefly, rat TA muscles were fixed in 4% paraformaldehyde at room temperature. Subsequently, TA muscles were flash-frozen in embedding medium, and sectioned on cryostat with 10-μm thickness. The cryosections were placed on glass slides. After blocking and washing, the slides were incubated for 12 h at 4°C with anti-laminin antibody (1:200; Abcam, Cambridge, United Kingdom). Sections were subsequently incubated with the Alexa Fluor secondary antibody (1:400; Invitrogen Antibodies, Waltham, MA, United States) for 30 min at room temperature. After washing, CSAs of myofibers were determined through a blinded analysis with the ImageJ software (NIH, Bethesda, MD, United States) of five randomly captured muscle images from each experimental condition.

### Enzyme-Linked Immunosorbent Assay

ELISA plates (Beyotime, Haimen, China) were washed with wash buffer for five times. The 100 μL muscle lysates from the TA muscles were incubated for 120 min at 37°C. Following, ELISA plates were washed and incubated with biotinylated polyclonal anti-rat IL6 antibody for 60 min at 37°C. Subsequently, ELISA plates were washed and incubated with HRP-Streptavidin for 20 min at 37°C in dark. Finally, enzyme activity was measured using TMB at 450 nm.

### Transmission Electron Microscopy (TEM) Analysis

To observe the changes of mitochondria, TA muscle was analyzed through TEM analysis. Briefly, one cubic millimeter-sized muscle was fixed in 2.5% glutaraldehyde followed by postfixation in 1% osmium tetroxide. Muscle images were collected by TEM (HT7700, Hitachi, Tokyo, Japan). A total of 20 fields per rat performed in three rats per condition were analyzed.

### Statistical Analysis

All data are presented as means ± SD. Results were analyzed using the *t* test when results from two experimental groups were compared or using One-way ANOVA when data from three or more groups were studied. All statistical analyses were conducted with a SPSS Software Version 17.0 (SPSS Inc., Chicago, IL, United States). *P* < 0.05 was considered statistically significant.

## Results

### Denervation Induces Pro-inflammatory Cytokine Expression in Skeletal Muscle

**FIGURE 1:**
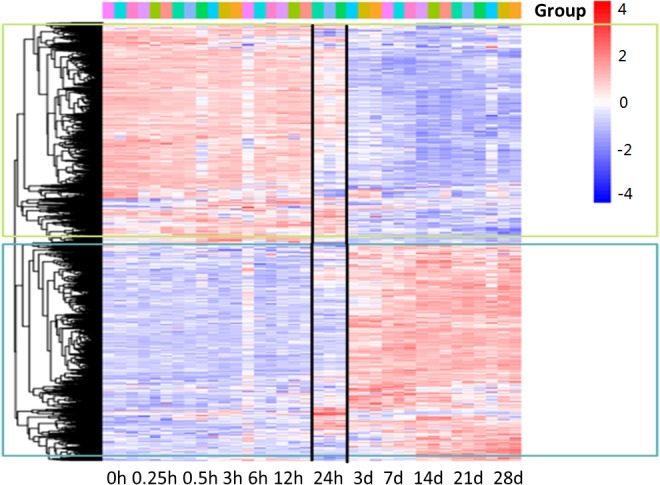
Gene-expression analysis during denervation-induced muscle atrophy. Heatmap showing distinct expression profiles of DEGs during tibialis anterior muscles atrophy induced by denervation. Blue and red indicate lower and higher transcript abundance, respectively. Bar at the top right corner represents log2 transformed values.

Microarray was used to analyze the differentially expressed genes during denervation-induced muscle atrophy. A total of 6581 differentially expressed genes (DEGs) were identified during denervation-induced tibialis anterior muscles atrophy. Interestingly, differential gene cluster analysis demonstrated that the DEGs were separated into two main profiles. One profile displayed a decrease with time, especially at 24 h following sciatic nerve injury. The other profile displayed an opposite trend with time, decreasing especially at 24 h following sciatic nerve injury. These data suggested that 24 h following sciatic nerve transection might be the key time points, as evidenced by the majority of DEGs from the two profiles began to appear at 24 h following sciatic nerve transection (Figure 1). Therefore, the DEGs at 24 h following sciatic nerve injury have become the focus of our attention.

**FIGURE 2:**
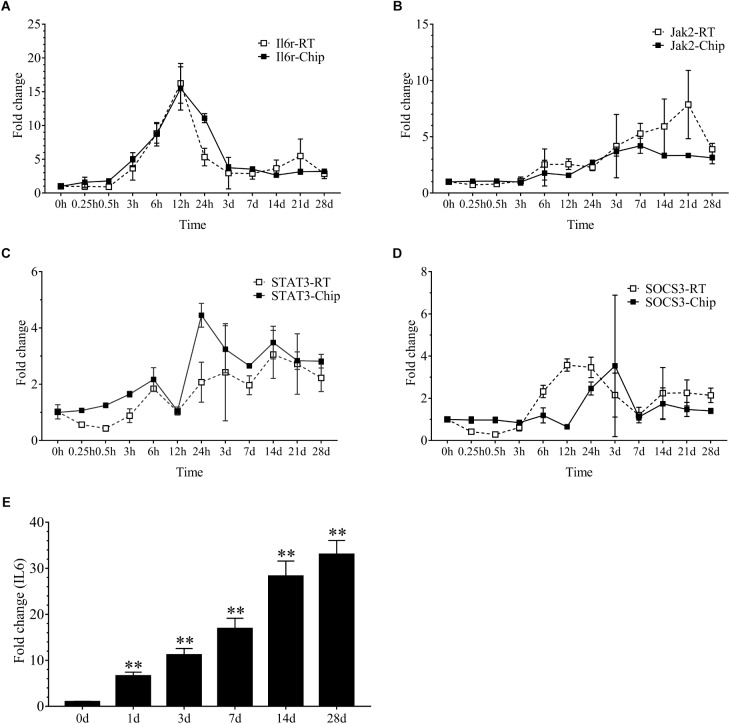
The inflammation-related factors were induced significantly during denervation-induced muscle atrophy. **(A–D)** The expression of inflammation-related factors (IL6r, Jak2, STAT3 and SOCS3) was examined during denervation-induced muscle atrophy. Hollow Square and Solid Square indicate the results from RT-qPCR validation and microarray analysis, respectively. **(E)** The content of IL6 was determined by ELISA in tibialis anterior (TA) muscle during denervation-induced muscle atrophy (^∗∗^*P* < 0.01 versus 0 d; *n* = 6).

Our results demonstrated that 2086 transcripts were differentially expressed at 24 h following sciatic nerve injury. There were 497 transcripts associated with inflammation. These transcripts mainly included *IL6r, Stat3, Jak2, Socs3, Cebpb, Tnfrsf1a, Myd88, Serpina3, Kras, Junb, IL1b* and so on. Interestingly, the vast majority of these inflammatory genes were up-regulated during denervation-induced muscle atrophy, which was further confirmed by the real time RT-PCR (Figure 2A–D). The up-regulation of receptor of *IL6 (IL6r)* was observed during denervation-induced muscle atrophy. Therefore, the expression of IL6 in TA muscles was determined by ELISA, and the results showed that the content of IL6 increased significantly during denervation-induced muscle atrophy (Figure 2E). In summary, denervation induced a large number of inflammatory cytokines in target muscles, especially for IL-6/STAT3 signaling pathways.

### IL6 Induces Muscle Atrophy and STAT3 Activation

**FIGURE 3:**
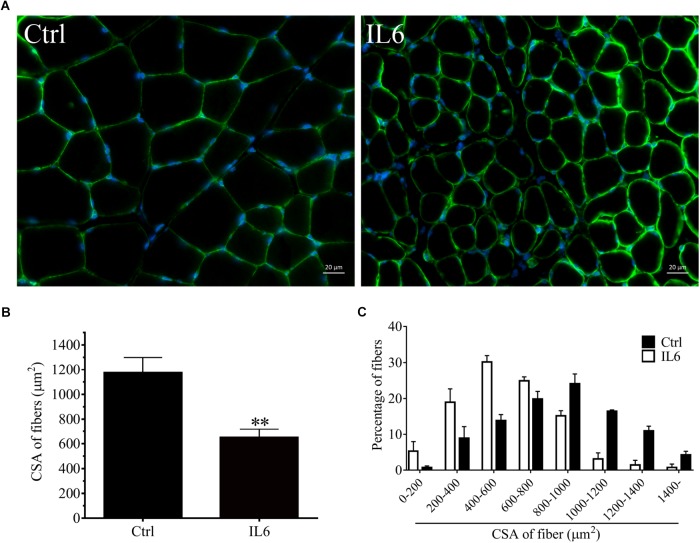
The muscle atrophy was induced by IL6 infusion in TA muscles of rats. After TA muscles of rats had been injected with saline vehicle or saline vehicle plus IL6 for 14 days, the TA muscles were harvested to undergo laminin staining analysis. Different muscle samples were harvested from rats receiving saline treatment (Ctrl, serving as normal control) and rats receiving IL6 infusion (IL6). **(A)** TA muscles of rats were stained for laminin after 14 d of saline or IL6 infusion. **(B)** Mean ± SEM of CSA in TA fibers from each group of rats (^∗∗^*P* < 0.01 for IL6-infused versus PBS-infused rats). **(C)** Frequency distribution of CSA in TA muscles of PBS or IL6-infused rats.

**FIGURE 4:**
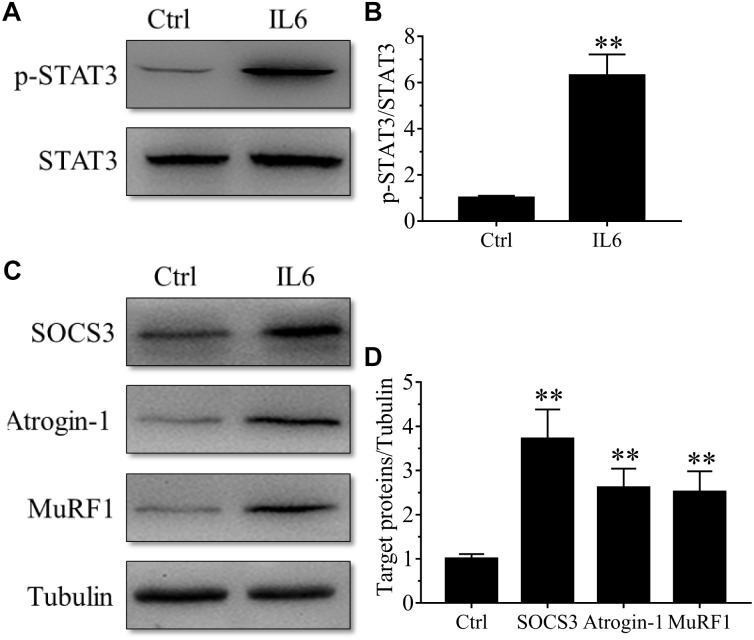
The STAT3/SOCS3 pathway and ubiquitin-proteasome system were induced by IL6 infusion for 14 days in TA muscles of rats. After TA muscles of rats had been injected with saline vehicle or saline vehicle plus IL6 for 14 days, the TA muscles were harvested to undergo Western blotting analysis. Different muscle samples were harvested from rats receiving saline treatment (Ctrl, serving as normal control) and rats receiving IL6 infusion (IL6). Representative Western blot images were shown for STAT3 phosphorylated at Tyr705 (P-STAT3) **(A)**, SOCS3, MuRF-1, and atrogin-1 **(C)**. Histograms comparing p-STAT3 **(B)**, SOCS3, MuRF-1, and atrogin-1 **(D)** levels among different muscle samples (*n* = 6). Results are the means ± SEMs. ^∗∗^*p* < 0.01 versus Ctrl.

To model the sustained high levels of IL6 observed in muscles, we implanted osmotic pumps delivering recombinant murine IL6 in normal rattus norvegicus. Administration of recombinant murine IL6 led to muscle atrophy. The mean CSA in TA muscles of the rats infused with IL6 was smaller than that in PBS-infused rats, and the frequency distribution of CSA of fibers in TA muscles of the rats infused with IL6 was shifted toward smaller sizes compared with results of saline infusion (Figure 3). At the same time, the expression of p-STAT3 displayed a significant increase in tibialis anterior muscles of rats administrated with IL6, accompanied by elevated expression of the proteins for STAT3 target genes SOCS3 and the two muscle-specific E3 ubiquitin ligase Atrogin-1/MAFbx and MuRF1 (Figure 4). Thus, it can be seen that IL6 alone is sufficient to induce STAT3 activation and muscle atrophy.

### Salidroside Attenuates Denervation-Induced Muscle Atrophy and Mitophagy and Inhibits the Production of IL6

**FIGURE 5:**
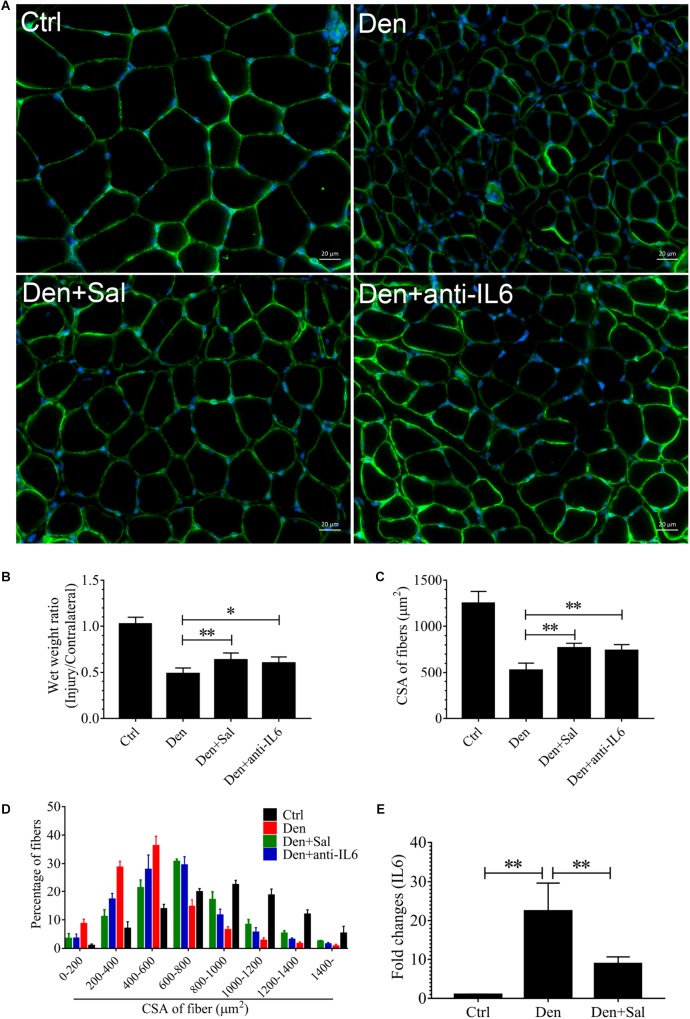
The muscle atrophy induced by denervation is significantly suppressed in Sal-infused and anti-IL6-infused rats. After denervation, TA muscles of rats had been injected with saline vehicle plus salidroside (Den+Sal), saline vehicle plus anti-IL6 (Den+anti-IL6) or saline vehicle only (Den) for 14 days. After sham operation, TA muscles of rats had been injected with saline vehicle (Ctrl) for 14 days. Then, the TA muscles were harvested to undergo laminin staining analysis. **(A)** TA muscles of rats were stained for laminin after 14 d of saline vehicle, salidroside or anti-IL6 infusion. **(B)** The wet weight ratio of TA muscles of rats was examined. **(C)** Mean ± SEM of CSA in TA fibers from each group. **(D)** Frequency distribution of CSA in fibers of TA muscles from saline vehicle, salidroside or anti-IL6-infused rats. **(E)** The content of IL6 in TA muscles from saline vehicle or salidroside (^∗^*P* < 0.05, ^∗∗^*P* < 0.01).

**FIGURE 6:**
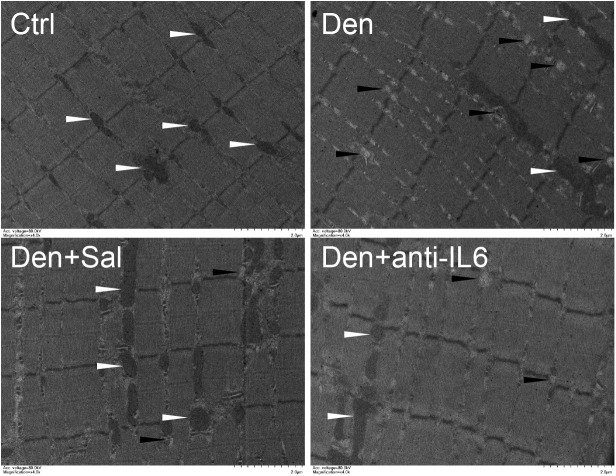
Salidroside inhibiting mitophagy might be through inhibiting IL6 in muscle. Representative TEM images of TA muscles treated with salidroside and anti-IL6. White arrow indicates normal mitochondria between muscle fibers. The black arrow indicates an autophage or an autophagic vesicle. A total of 20 fields per rat were analyzed (*n* = 3 rats in each group).

To examine whether salidroside plays a role in denervation-induced muscle atrophy, we compared denervated rats administrated with salidroside or saline. After 14 d of salidroside infusion, the denervation-induced loss of muscle mass was significantly blocked in the rats administrated with salidroside. Likewise, the mean CSA in TA muscles of the denervated rats infused with salidroside was higher than that in saline-infused denervated rats, and the frequency distribution of CSA of fibers in TA muscles of the denervated rats infused with salidroside was shifted toward bigger sizes compared with results of saline infusion (Figure 5). Furthermore, salidroside inhibited mitophagy (Figure 6). At the same time, compared with TA muscles of the denervated rats infused with saline, the content of IL6 reduced remarkably in TA muscles of the denervated rats infused with salidroside (Figure 5). The effects of salidroside on denervated muscles were also observed in the denervated muscles administrated with anti-IL6 (Figure 5, 6). These results uncover a new role for salidroside.

### Salidroside Inhibiting Proteolytic Pathways Might Be Through Inhibiting IL6

**FIGURE 7:**
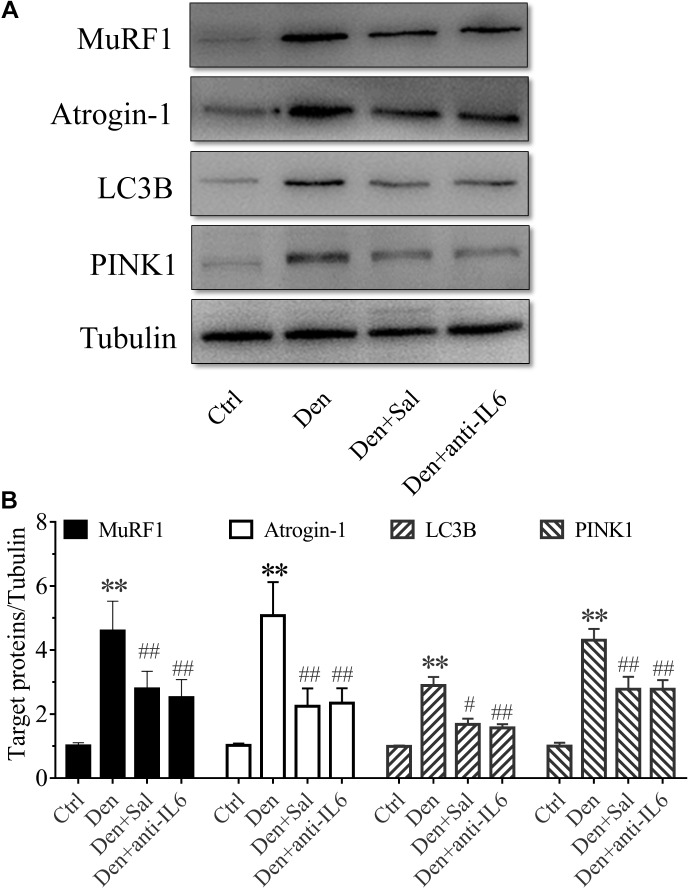
Salidroside inhibiting proteolytic pathways might be through inhibiting IL6 in muscle. **(A)** Representative blots of Atrogin-1, MuRF1, LC3B and PINK1 in TA muscles treated with salidroside and anti-IL6. **(B)** Relative expression of Atrogin-1, MuRF1, LC3B and PINK1 in TA muscles treated with salidroside and anti-IL6 (*n* = 6 rats in each group). ^∗∗^*p* < 0.05 versus Ctrl. ^#^*p* < 0.05 and ^##^*p* < 0.01 versus Den.

To assess how salidroside attenuates muscle atrophy, we examined evidences for the ubiquitin proteasome system (UPS) and autophagy lysosome pathway (ALP) proteolytic activities. The expression of muscle-specific E3 ubiquitin ligases Atrogin-1/MAFbx and MuRF1 were suppressed by salidroside in denervated muscles (Figure 7). There also was a decrease in the expression of the ALP related proteins LC3B and PINK1 (Figure 7). Likewise, these responses to salidroside infusion were also observed in the muscles of anti-IL6 infusion (Figure 7). These data suggested that salidroside inhibiting proteolytic pathways might be through inhibiting IL6.

### Salidroside Suppressing p-STAT3 and SOCS3 Expression Might Be Through Inhibiting IL6

**FIGURE 8:**
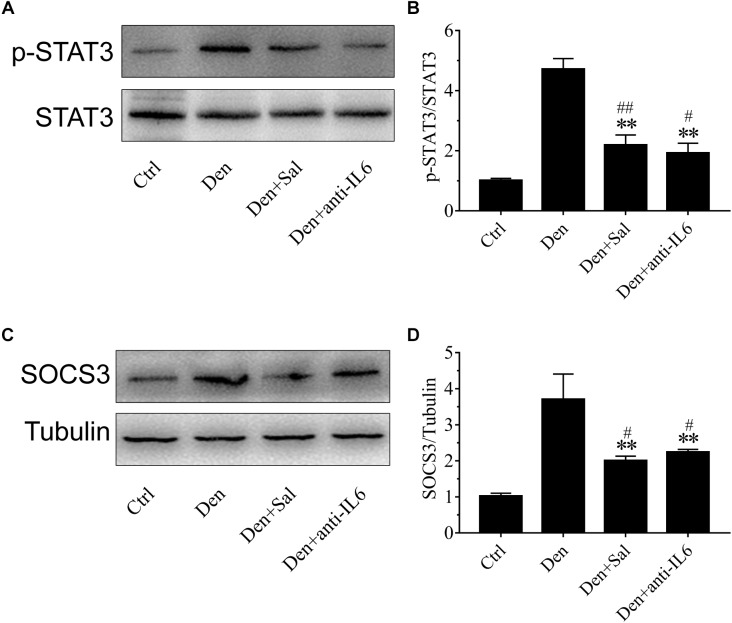
Salidroside suppressing p-STAT3 and SOCS3 expression might be through inhibiting IL6 in muscle. **(A,C)** Representative blots of p-STAT3 and SOCS3 in TA muscles treated with salidroside or anti-IL6. Salidroside decreased the expression of p-STAT3 and SOCS3 in the denervated muscles. **(B,D)** Relative expression of p-STAT3 and SOCS3 in TA muscles treated with salidroside or anti-IL6. Anti-IL6 decreased the expression of p-STAT3 and SOCS3 in the denervated muscles (*n* = 6 rats in each group). ^∗∗^*p* < 0.01 versus Den. ^#^*p* < 0.05 and ^##^*p* < 0.01 versus Ctrl.

To understand how salidroside downregulates proteolytic pathways, we evaluated STAT3/SOCS3 signaling. SOCS3 is one of STAT3 target genes, which led to impairing insulin/IGF-1 signaling and enhancing protein degradation (Zhang et al., 2009). In salidroside-infused denervated rats, phosphorylated STAT3 levels in muscles were sharply decreased (Figure 8A). SOCS3 protein in the muscles of salidroside-infused denervated rats displayed a significant decrease (Figure 8B). Interestingly, these responses to salidroside infusion were also observed in the muscles of anti-IL6 infusion. The phosphorylated STAT3 and SOCS3 levels displayed significant downregulation in the muscles administrated with anti-IL6 (Figure 8). In summary, we might speculate that salidroside suppressing p-STAT3 and SOCS3 expression might be through inhibiting IL6.

## Discussion

Skeletal muscle homeostasis is maintained by the equilibrium of physical and functional interactions between myofibres and muscle microenvironment, which includes muscle stem cells, motoneurons, interstitial cells and a variety of secretory factors (Madaro et al., 2018). Efforts to elaborate the underlying mechanisms of muscle atrophy have been predominantly focused on intrinsic events of myofibres. On the contrary, less attention has been paid to potential contributory factors (e.g., inflammatory cytokines) within the muscle microenvironment (He et al., 2013; Saini et al., 2016). When muscle microenvironment is subjected to perturbations, it could trigger muscle atrophy (Talbert and Guttridge, 2016). Consequently, can we prevent muscle atrophy from protecting muscle microenvironment? To our knowledge, the current study is the first one to demonstrate that salidroside could improve muscle microenvironment to attenuate the skeletal muscle atrophy induced by denervation in adult rats.

Microarray analysis demonstrated that 24 h might be the key time points for the initiation of muscular atrophy, as evidenced by the majority of DEGs began to appear at 24 h following sciatic nerve transection. It also suggested that the treatment time of denervated muscle atrophy had better be within 24 h of nerve injury, which may inhibit the onset of muscle wasting. Our results also demonstrated that denervation induced a large number of inflammatory cytokines in target muscles, especially for IL6/STAT3 signaling pathways, and that IL6 content displayed a significant upregulation in muscles after nerve injury. Evidences have suggested that elevated IL-6 levels in most experimental models of cachexia are responsible at least in part for cachexia-induced muscle atrophy (Bonetto et al., 2012). To identify whether IL6 plays a crucial role in denervation-induced muscle atrophy, we initially implanted osmotic pumps delivering recombinant murine IL6 in normal rats, and found that IL6 alone was sufficient to induce STAT3 activation and muscle atrophy. This is consistent with previous study that exogenous IL6 can induce cachexia and muscle wasting (Zimmers et al., 2016). This process is actually based on the interaction between IL6 and IL6 receptors because blocking IL6 receptor could inhibit muscle wasting in mice that overexpress IL6 (Goodman, 1994; Fujita et al., 1996; Tsujinaka et al., 1996). These results suggested that denervation destroyed muscle microenvironment and induced a large number of inflammatory cytokines, such as IL6, which was sufficient to cause muscle atrophy. Consequently, we wondered whether inhibiting inflammation and improving muscle microenvironment could effectively inhibit denervation-induced muscle atrophy.

Salidroside is a biologically active ingredient of Rhodiola rosea, which exhibits anti-inflammatory properties (Lai et al., 2015; Sun et al., 2018). To examine whether salidroside plays a role in improving the muscle microenvironment after nerve injury and attenuates denervation-induced muscle atrophy, we compared denervated rats administrated with salidroside or saline. After 14 d of salidroside infusion, the denervation-induced loss of muscle mass and decreased CSA were significantly blocked, and the content of IL6 reduced remarkably in TA muscles of the denervated rats infused with salidroside. These data demonstrated that salidroside inhibited denervation-induced muscle atrophy and the production of IL6 in denervated muscles. The effects of salidroside on denervated muscles were similar to that observed in the denervated muscles administrated with anti-IL6. Evidences have demonstrated that salidroside attenuated the infiltration of inflammatory cells and decreased the levels of serum IL6 obviously in LPS induced inflammation (Li et al., 2013; Yan and Choi, 2014; Qi et al., 2016; Wu et al., 2016). Salidroside also reduced the expression of IL6 in serum, and then exerts strong favorable cardioprotective function on myocardial I/R injury (Zhu et al., 2015). These results suggested that salidroside protected against denervation-induced muscle atrophy, which may be caused by the inhibition of proteolytic pathways due to salidroside-induced decrease in IL6.

The ubiquitin-proteasome pathway and autophagy-lysosomal pathway are two main intracellular proteolysis systems, serving as major proteolytic machineries in different muscle atrophy models (Polge et al., 2015; Wing, 2016; Qiu et al., 2018). To assess how salidroside attenuates muscle atrophy, we examined the activities of ubiquitin proteasome system and autophagy lysosome pathway. Current study showed that ubiquitin proteasome system (Atrogin-1/MAFbx and MuRF1) and autophagy lysosome pathway (LC3B and PINK1) were remarkably suppressed by salidroside in denervated muscles. That is to say that salidroside could suppress the activities of intracellular proteolysis systems. At the same time, salidroside decreased the content of IL6 in denervated muscles. Inflammation has been associated with activation of proteolysis systems in muscle (Zhang et al., 2009). Zimmers et al. (2016) found that IL-6 inhibition could reduce muscle loss in cancer. Likewise, current study demonstrated that anti-IL6 infusion only also inhibited the proteolytic activities and muscle wasting induced by denervation. Therefore, these data suggested that salidroside inhibited proteolytic pathways via inhibiting IL6.

A trigger of muscle protein loss is STAT3, an IL6–activated transcription factor stimulating SOCS3 expression (Zhang et al., 2006). The activation of STAT3 is crucial for the activation of proteolytic pathways in muscle atrophy caused by different types of cancer and sterile sepsis (Bonetto et al., 2012; Silva et al., 2015; Ma et al., 2017). To understand how salidroside downregulates proteolytic pathways, we evaluated STAT3/SOCS3 signaling. Current study showed that salidroside suppressed p-STAT3 and SOCS3 expression. It has been reported that salidroside can inhibit the expression of p-STAT3 in various diseases (Lv et al., 2016; Qi et al., 2016; Li and Chen, 2017). Li et al. demonstrated that the p-STAT3 could be suppressed by salidroside in many cancers (Lv et al., 2016; Li and Chen, 2017). Salidroside also inhibited the activation of JAK2-STAT3 pathway induced by LPS (Qi et al., 2016). Silva et al. (2015) found that p-STAT3 stimulated muscle protein losses through activation of proteolytic pathways and that blocking p-STAT3 with C188-9, STAT3 inhibitor, could prevent loss of muscle cell proteins through suppressing proteolytic pathways. From this we can speculate that salidroside suppressing the proteolytic pathways might be through inhibiting p-STAT3. SOCS3 is one of STAT3 target genes. Our data showed that salidroside also suppressed SOCS3 expression. The increase in SOCS3 led to impairing insulin/IGF-1 signaling and enhancing protein degradation (Zhang et al., 2006, 2009). IL6 could promote IRS-1 degradation by upregulating SOCS3 in muscle (Zhang et al., 2009). Current study also suggested that blocking IL6 could decrease the phosphorylated STAT3 and SOCS3 levels in muscles. In summary, we might speculate that salidroside downregulates proteolytic pathways through suppressing p-STAT3 and SOCS3 expression.

**FIGURE 9:**
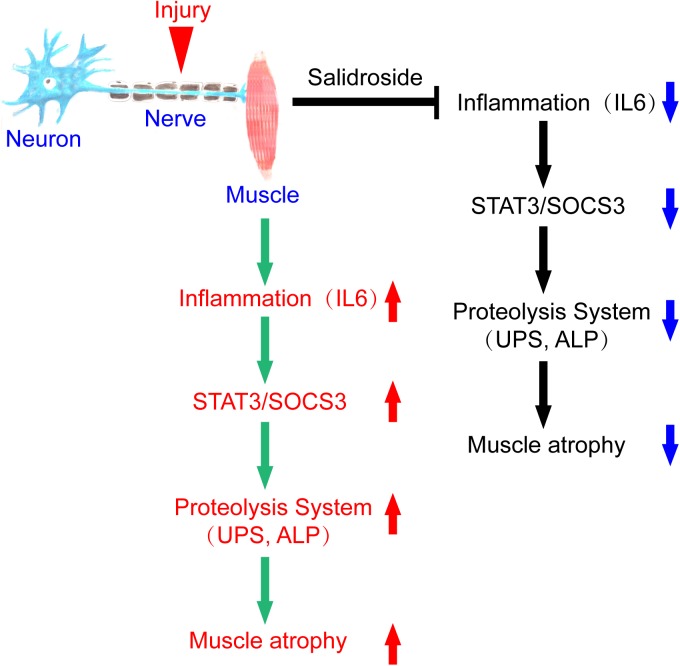
A scheme for salidroside attenuating denervation-induced muscle atrophy by inhibiting the activation proteolytic pathways through the blocking of IL6/STAT3/SOCS3 signaling pathways.

In summary, we conclude that denervation induces inflammation and stimulates inflammatory cytokines production, especially IL6, in muscles, which increases STAT3 phosphorylation and SOCS3 expression. The result is activation of proteolytic pathways in muscles. Salidroside alleviates denervation-induced inflammation response, thereby inhibits muscle proteolysis and muscle atrophy. This process might be via inactivating STAT3/SOCS3 pathways (Figure 9). It follows that salidroside might be a potential therapeutic candidate to prevent muscle wasting.

## Data Availability

This manuscript contains previously unpublished data. The gene expression dataset is available on EBI and the accession number is E-MTAB-8009.

## Ethics Statement

This study was carried out in accordance with the recommendations of the Institutional Animal Care and Use Committee of Nantong University. The protocol was approved by the Institutional Animal Care and Use Committee of Nantong University.

## Author Contributions

HS designed the study. CW, LT, TX, LX, WM, and XY performed the experiments. CW, XN, QF, and WM collected and assembled the data. CW, XN, LT, and WM performed the data analysis. HS provided scientific expertise. HS and CW wrote the manuscript.

## Conflict of Interest Statement

The authors declare that the research was conducted in the absence of any commercial or financial relationships that could be construed as a potential conflict of interest.
